# Public interest in different types of masks and its relationship with pandemic and policy measures during the COVID-19 pandemic: a study using Google Trends data

**DOI:** 10.3389/fpubh.2023.1010674

**Published:** 2023-06-08

**Authors:** Andy Wai Kan Yeung, Emil D. Parvanov, Jarosław Olav Horbańczuk, Maria Kletecka-Pulker, Oliver Kimberger, Harald Willschke, Atanas G. Atanasov

**Affiliations:** ^1^Oral and Maxillofacial Radiology, Applied Oral Sciences and Community Dental Care, Faculty of Dentistry, The University of Hong Kong, Hong Kong, China; ^2^Ludwig Boltzmann Institute Digital Health and Patient Safety, Medical University of Vienna, Vienna, Austria; ^3^Department of Translational Stem Cell Biology, Research Institute of the Medical University of Varna, Varna, Bulgaria; ^4^Institute of Genetics and Animal Biotechnology of the Polish Academy of Sciences, Magdalenka, Poland; ^5^Institute for Ethics and Law in Medicine, University of Vienna, Vienna, Austria; ^6^Department of Anaesthesia, Intensive Care Medicine and Pain Medicine, Medical University Vienna, Vienna, Austria

**Keywords:** public health, SARS-CoV-2, COVID-19, Coronavirus, face mask, surveillance, pandemic, digital health

## Abstract

Google Trends data have been used to investigate various themes on online information seeking. It was unclear if the population from different parts of the world shared the same amount of attention to different mask types during the COVID-19 pandemic. This study aimed to reveal which types of masks were frequently searched by the public in different countries, and evaluated if public attention to masks could be related to mandatory policy, stringency of the policy, and transmission rate of COVID-19. By referring to an open dataset hosted at the online database Our World in Data, the 10 countries with the highest total number of COVID-19 cases as of 9th of February 2022 were identified. For each of these countries, the weekly new cases per million population, reproduction rate (of COVID-19), stringency index, and face covering policy score were computed from the raw daily data. Google Trends were queried to extract the relative search volume (RSV) for different types of masks from each of these countries. Results found that Google searches for N95 masks were predominant in India, whereas surgical masks were predominant in Russia, FFP2 masks were predominant in Spain, and cloth masks were predominant in both France and United Kingdom. The United States, Brazil, Germany, and Turkey had two predominant types of mask. The online searching behavior for masks markedly varied across countries. For most of the surveyed countries, the online searching for masks peaked during the first wave of COVID-19 pandemic before the government implemented mandatory mask wearing. The search for masks positively correlated with the government response stringency index but not with the COVID-19 reproduction rate or the new cases per million.

## Introduction

The COVID-19 pandemic that began at the end of 2019 has continued to affect the global population. Since 2022, the highly contagious Omicron variant has become the dominant type (1). Many countries had consistently high face mask usage or transitioned from low to high usage (2). Some researchers advocated for more well-designed and robust experiments before using research findings to inform policy on mask wearing (3). Indeed, a randomized controlled trial reported that the recommendation to wear surgical masks to supplement other public health measures did not reduce the COVID-19 infection rate by more than 50% in a Danish community (4). On the other hand, another randomized controlled trial found that mask distribution and promotion could significantly increase mask-wearing rate and reduce symptomatic COVID-19 cases in a Bangladesh rural district (5). Readers can refer to two recent review papers that summarized the findings from clinical trials and experiments that demonstrated the efficacy of masks in reducing the transmission of respiratory viruses (6, 7).

At the beginning of the COVID-19 pandemic the mostly used masks as protective wear were cloth face masks and surgical masks, both of which were available in higher quantities and more amendable for rapid production-upscale (8). Later with the ongoing course of the COVID-19 pandemic and increased medical industry production, higher level protection masks such as N95, KN95 and FFP2 were introduced. These three types of masks have filtration capacity of over 94% and have different market distribution based on their certification (FFP2—European-certified, KN95—Chinese-certified, N95—American-certified). In addition, masks with even higher efficiency (> 98% filtration capacity) were produced (FFP3). Overall, the sophisticated production of the higher level protection masks led to shortage in their distribution and even an export ban by some producing countries during the COVID-19 pandemic (9).

Implementing a mandatory policy was found to increase mask wearing compliance during the early phase of the COVID-19 pandemic, for example in Germany (10). In order to help and support the mandatory masks wearing, a number of countries removed or reduced the value-added tax from the masks' price. Meanwhile, Google Trends data reflect the online searches for a particular term or topic by the public. Using Google Trends to track the search for masks in the last few years can reveal the public responses to COVID-19. It was demonstrated that the search volume for surgical masks from Google Trends significantly correlated with their actual online sales volume and hence a “good surrogate to reflect public interest” (11). Google Trends is a feasible source to acquire data regarding public interest, since online searching is a daily routine for a majority of the public, and Google Trends offers open and free data reflecting searching behaviors. In this context, Google Trends has been used to investigate various themes on online information seeking during the COVID-19 pandemic era (12–16). Several studies that investigated Google Trends data on mask searching during the COVID-19 pandemic have been published with different research foci and research gaps (Table 1). None of them obtained data from multiple countries for a long time period. As a matter of fact, a Dutch study found that both the implementation of smoke-free legislation and reimbursement of smoking cessation support were associated with a 16–41% increase in relative search volume (RSV) of Google searches for “quit smoking” in the short-to-medium term (20). Such relationship was not tested between mask policy and Google search for masks. As different countries implemented stringent mask policy at different times, it was expected that such relationship might be observed for various countries.

**Table 1:** Prior studies using Google Trends data on mask searching during the COVID-19 pandemic.

**References**	**Location and time frame**	**Findings and conclusions**	**Research gaps**
Anwar et al. (17)	Algeria, March 2020	RSV for masks had a positive correlation with the number of daily cases during the time frame of 1–14 March (14 days) to 1–19 March (19 days). A similar positive correlation was observed with the number of daily deaths during the time frame of 1–17 March (17 days) to 1–19 March (19 days).	Data was obtained from one country for 1 month only.
Wong et al. (11)	Global data and 42 individual countries/regions, January to May 2020.	Global RSV for masks had a positive correlation with Amazon and eBay sales volume of masks over a three-month time frame (January to March 2020). Country RSV for masks had a negative correlation with average number of daily cases during the same period.	Data was obtained during the initial phase of COVID-19 pandemic only.
Goldberg et al. (18)	United States, 3–7 April 2020	RSV for masks reached its peak on 4 April, one day after the Centers for Disease Control and Prevention (CDC) recommended all Americans to wear masks. RSV then decreased sharply.	Data was obtained from one country for 5 days only.
Lang et al. (19)	United States, March to August 2020	RSV for masks reached its peak in April, then decreased sharply, and finally rebounded to a smaller 2nd peak in July.	It did not test if RSV data was related to COVID-19 data.

It would be interesting to know which types of masks were sought after by the public in different countries, and if public attention to masks could be related to mandatory policy, stringency of the policy, and transmission rate of COVID-19 in the long run. This study aimed to explore Google Trends data to provide insights addressing the above-listed research gaps on which no previous information existed in the scientific literature.

## Methods

The open and actively updated dataset named Coronavirus (COVID-19) Vaccinations (21), hosted by the Our World in Data (OWID), was accessed on 9th of February 2022. The top 10 countries with highest number of total confirmed cases were selected, namely (in descending order) the United States, India, Brazil, France, United Kingdom, Russia, Turkey, Italy, Germany, and Spain. Coincidentally, at this time point they were the only countries with >10 million total cases each. For each of these countries, the weekly new cases per million population, reproduction rate (of COVID-19), stringency index, and face covering policy score were computed. The entire available OWID dataset was downloaded, and its earliest data available could be traced back to 5th of January 2020, depending on country and metric. Basically, all four metrics were provided by the OWID database on a daily basis, and we computed the daily data into weekly data by averaging the data from every 7 days. For instance, the weekly metric for the week of 5th of January 2020 was computed as the average of the 7-day data from 5th to 11th of January 2020. If there was some missing data for a certain country during a certain week, then we used the available data to compute the weekly data. For instance, if data from 1 day was missing in a week, then the data from the remaining 6 days was used to compute for the average; if data from all 7 days was missing in a week, then that weekly data was left as blank.

Next, the definitions or coding of the four metrics according to the OWID database were explained. According to the database, the stringency index was calculated by the Oxford Coronavirus Government Response Tracker (OxCGRT) project (22), and was “a composite measure based on 9 response indicators including school closures, workplace closures, and travel bans, rescaled to a value from 0 to 100, with 100 being the strictest.” Meanwhile, the face covering policy score reflected the strictness of the policy adopted in a specific country (0 = No policy; 1 = Recommended; 2 = Required in some shared/public spaces with other people present, or some situations when social distancing not possible; 3 = Required in all shared/public spaces with other people present or all situations when social distancing not possible; 4 = Required outside the home at all times regardless of location or presence of other people). Last but not least, the reproduction rate represented the “average number of new infections caused by a single infected individual.” The OWID database calculated this metric on a daily basis according to the statistical model introduced by Arroyo-Marioli et al. (23), which considered for the temporal changes in the number of susceptible, infected, and recovered individuals. If the rate is > 1, the infection would continue to spread out in the population. If it is <1, the number of cases in the population would gradually reduce to zero.

Then, Google Trends were queried to extract the RSV data from each of these countries. The queries were limited to the period from the 1st of January 2020 to the 9th of February 2022. Google Trends output weekly data that began from 5th of January 2020 (i.e., data from the 1st week was from 5th to 11th January 2020) and therefore the weekly data from OWID was similarly computed. The default “All categories” and “Web search” were selected. First, 4 types of masks were considered, namely N95, FFP2, surgical, and cloth face mask. Initially, additional types were considered for potential inclusion, such as KN95, KF94, N99, ASTM Level 3, and FFP3. However, Google Trends could only search the indicated first 4 types of masks as “topics” and the following additional types of masks as “search terms.” Comparing the RSV data between topics and search terms would produce bias, as the former basically includes all search terms related to it, whereas the latter is specific. Hence, only the mask types designated as “topics” were considered to evaluate their relative popularity in terms of RSV in each country. In the end, 4 “topics” were searched for each country, namely: “Particulate Respirator Type N95,” “FFP2 mask,” “Surgical mask,” and “Cloth face mask.” Different from “search terms” that can be entered with any words, these topic names were defined by Google Trends by default. Supplementary Figure 1 illustrates the search process in Google Trends with the N95 mask used as an example.

Second, mask as a topic was queried for each country. This data would be plotted with the OWID data described above, so that readers could see if the mask searching volume covaried with COVID-19 metrics in each country.

For statistical analysis, Spearman's correlation tests were conducted to evaluate the relationship between Google Trends RSV of mask as a topic and three COVID-19 metrics, namely reproduction rate, stringency index, and new cases per million. To test for time-lag between RSV of mask as a topic and new cases per million, cross-correlation tests were performed with reference to The Odum Institute (24). For the “Transform” options, the “natural log transform” was checked, and the “difference” was set to be 1. Statistical tests were performed with SPSS (version 26.0; IBM, NY, USA). Results were deemed significant if *p* < 0.05.

## Results

**Figure 1:**
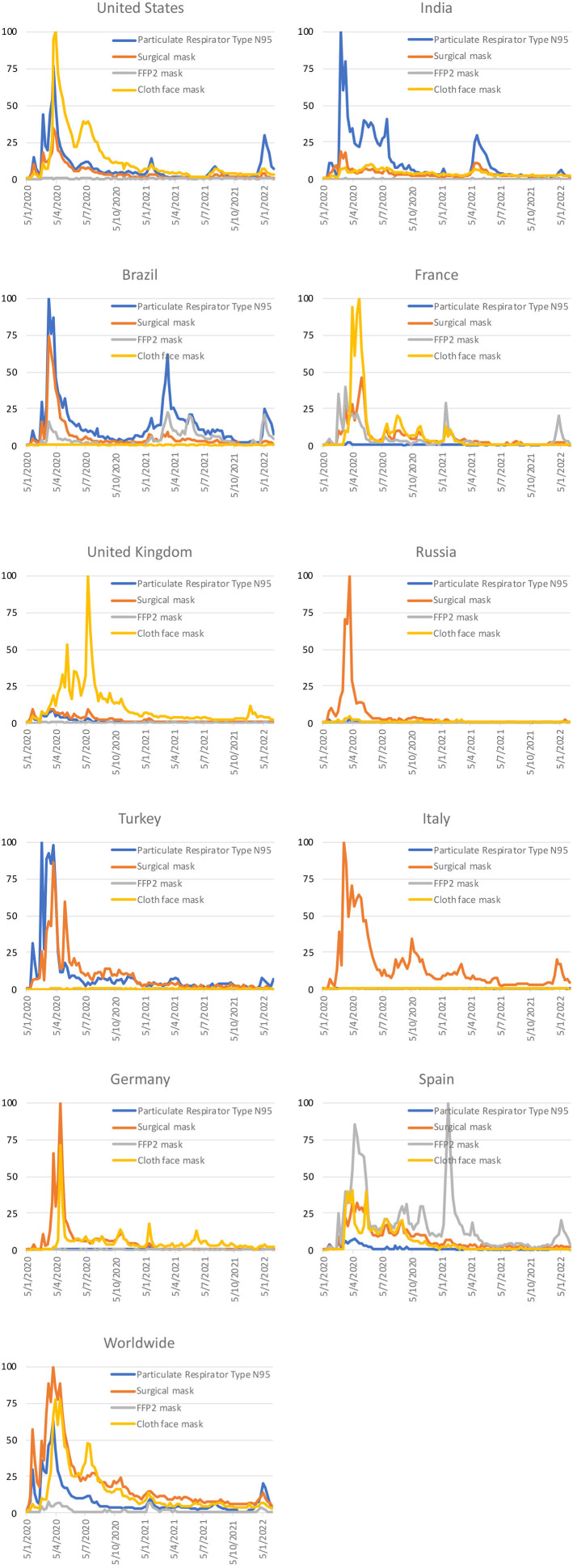
Google Trends relative search volume of the 4 mask types by different countries.

Figure 1 shows the Google Trends relative search volume (RSV) of the 4 masks in each of the top 10 countries with highest number of total confirmed cases. The data could be found in Supplementary material 1. Google searches for N95 were predominant in India, whereas surgical masks were predominant in Russia, FFP2 masks were predominant in Spain, and cloth masks were predominant in both France and United Kingdom. Google searches from the United States, Brazil, Germany, and Turkey had 2 predominant mask types. Nearly all countries had their RSV peaked in the first quarter of 2020, coinciding with the first wave of COVID-19 pandemic. Some European countries, such as France and Spain, had another boost in RSV for FFP2 masks in around January 2021 when some European countries made FFP2 masks (or other masks with high grade of protection) mandatory. When the infectious COVID-19 Omicron variant began to predominate in January 2022, a small boost in RSV of N95, surgical mask, or FFP2 mask (but not cloth mask) was observed in most of these countries.

### Relationship between mask searching and COVID-19 metrics

**Figure 2:**
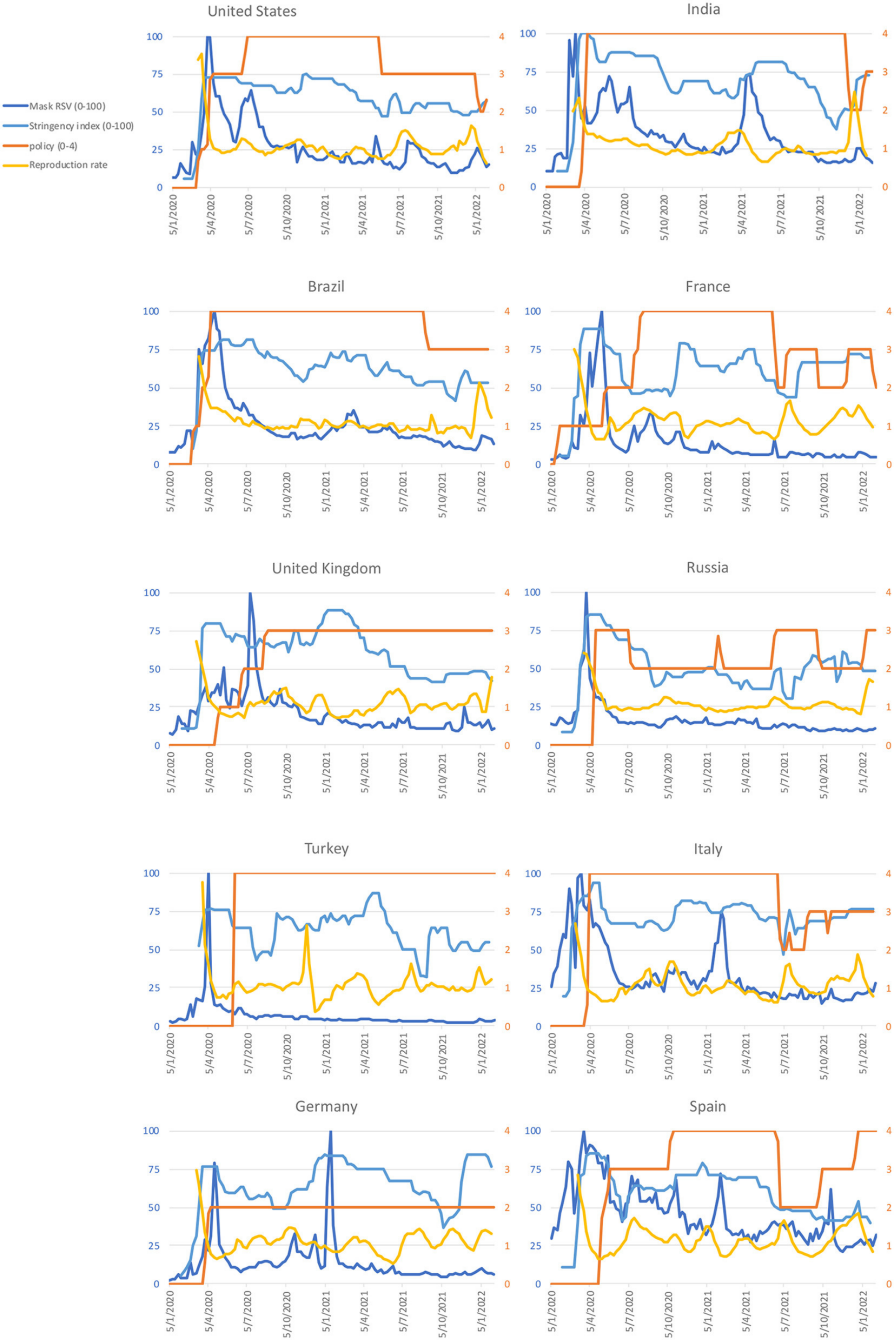
Temporal data of Google Trends (relative search volume, RSV, of mask as a topic) and COVID-19 metrics (details explained in the Methods). Mask RSV and stringency index are plotted on the left y-axis (blue), whereas policy score and reproduction rate are plotted on the right y-axis (orange).

**Figure 3:**
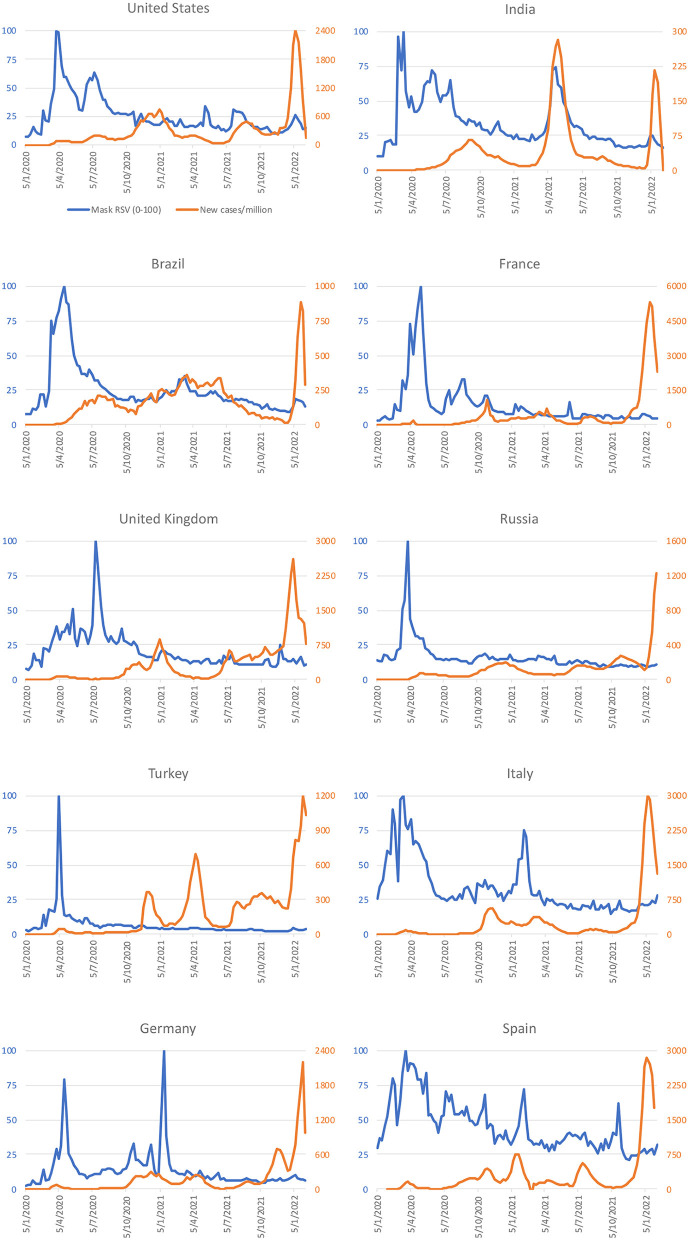
Temporal data of Google Trends (relative search volume, RSV, of mask as a topic) and COVID-19 new cases per million.

The RSV for masks peaked before the government implemented some form of mandatory face covering policy (policy score 2 or above in Figure 2) in 7 of the 10 countries, namely the United States, India, France, Russia, Turkey, Italy, and Spain (Figure 2). Mask RSV positively correlated with stringency index in 8 countries (Table 2), and positively correlated with COVID-19 reproduction rate in 2 countries (Table 2). Interestingly, it showed positive correlation with new cases per million population in 1 country, but showed a negative correlation in 5 countries. The mask RSV did not experience a particular increase in these 10 countries during the Omicron wave, though recorded high new cases per million was recorded (Figure 3).

**Table 2:** Spearman correlations between Google Trends (relative search volume, RSV, of mask as a topic) and COVID-19 metrics.

**Country**	**Reproduction rate**	***P*-value**	**Stringency index**	***P*-value**	**New cases per million**	***P*-value**
US	0.162	0.107	0.583	**7** **×10**^**−11**^	−0.176	0.069
India	0.326	**0.001**	0.684	**9** **×10**^**−16**^	0.192	**0.048**
Brazil	0.541	**7** **×10**^**−9**^	0.816	**5** **×10**^**−25**^	0.168	0.091
France	0.087	0.388	0.137	0.165	−0.273	**0.004**
UK	0.052	0.607	0.541	**3** **×10**^**−9**^	−0.492	**9** **×10**^**−8**^
Russia	0.097	0.341	0.099	0.312	−0.574	**1** **×10**^**−10**^
Turkey	0.065	0.525	0.425	**1** **×10**^**−5**^	−0.676	**1** **×10**^**−14**^
Italy	−0.048	0.635	0.281	**0.004**	−0.140	0.153
Germany	−0.190	0.059	0.280	**0.004**	−0.048	0.626
Spain	−0.033	0.748	0.354	**0.0002**	−0.391	**4** **×10**^**−5**^

Bolded *p*-values were significant (<0.05). UK, United Kingdom; US, United States.

**Figure 4:**
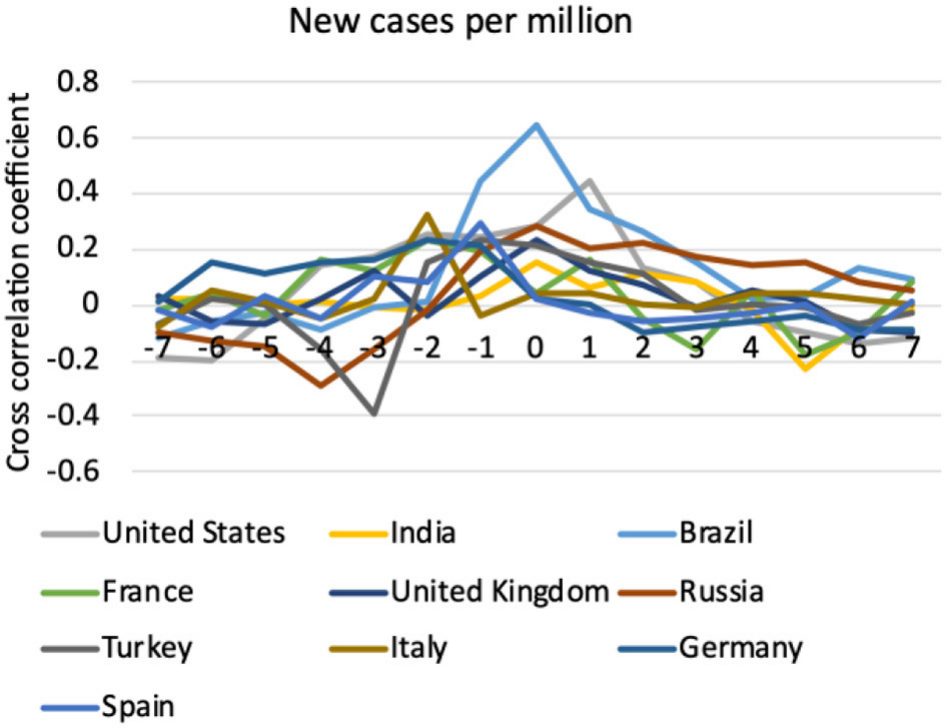
Cross correlation coefficient of Google Trends (relative search volume, RSV, of mask as a topic) with COVID-19 new cases per million, with natural log transform applied to both variables.

Figure 4 shows the cross-correlation results between RSV of mask as a topic and new cases per million. It could be observed that data from many countries had the highest coefficient at lag = 0, implying that their change in volume of online mask searching usually coincided with the change in new cases per million (*P* < 0.05 for Brazil, United Kingdom, Russia, and Turkey). Italy had the highest coefficient at lag = −2, meaning that change in volume of online mask searching preceded the change in new cases per million by 2 weeks. Meanwhile, the United States had the highest coefficient at lag = +1, meaning that change in volume of online mask searching lagged behind the change in new cases per million by 1 week.

## Discussion

On 6th of April 2020, World Health Organization (WHO) has updated its guidance to “advise that to prevent COVID-19 transmission effectively in areas of community transmission, governments should encourage the general public to wear masks in specific situations and settings as part of a comprehensive approach to suppress SARS-CoV-2 transmission” (25). It could be observed from our results that many countries' RSV of mask reached its highest peak on this occasion or slightly after this occasion, including the United States, Brazil, and Turkey. Meanwhile, some countries already reached their peak 1–3 weeks before this day, such as India, Russia, Italy, and Spain. In fact, regional governments might have issued a similar guidance or instruction before WHO's announcement. For instance, earlier on 19th of March 2020, California governor issued a statewide shelter-in-place order, requesting residents to remain at home and to wear a mask if they left their homes (26). In this study, Google Trends data revealed that populations in the 10 countries with highest number of confirmed COVID-19 cases had already intensively searched for online information regarding different types of face masks before this announcement. Similarly, the peak of the online search for masks preceded the implementation of official requirement to wear masks in public spaces in most of these countries. These pieces of information suggested that the public was relatively proactive in seeking online information on getting protection from face masks. The relative volumes of online search for masks positively correlated to the stringency of measures implemented by the governments. This meant that the public could potentially comply well to policy-makers, but that needed to be built upon high levels of public trust in government and the perception of its truthfulness, across different political regimes (27).

On the other side, the relative volumes of online search for masks did not seem to relate to the number of new cases per million or even showed a negative correlation. In the early phase of the COVID-19 pandemic (from late January 2020 until late May 2020), Wong et al. (11) reported that countries/regions with higher search volumes for masks had lower number of daily cases. Similarly, Feng et al. noticed that public interest (online search for mask via search engine Baidu) and risk perception had a more significant association with mask demand during the outbreak stage (28). While being proactive in wearing masks might reduce total infections and deaths due to COVID-19 (29), the current study revealed that the public no longer online-searched for masks as actively as before, when subsequent waves of infection arrived. This was obvious when the Omicron variant became predominant in the January 2022 causing record highs of new cases per million, the RSV of masks (and their various types) was only a small fraction of its peak recorded during the first wave in early 2020. Indeed, early phase data (from late January 2020 until early April 2020) in the United States showed that the Google Trends RSV of face mask peaked toward the end of the survey period (i.e., early April 2020) (30), consistent to the current results. One difference was that the online search for masks positively correlated with daily new COVID-19 cases in that early phase, but such relationship could not be observed across the longer period, regardless of United States or other countries, as analyzed in this study. One reason could be that the public has already got used to wearing masks with enough information with regards to their choices, functionality, benefits, drawbacks, and so on. Another reason could be related to “pandemic fatigue,” a notion that people had behavioral fatigue with adhering to the COVID-19 restrictions (31) and such fatigue could counteract their continued interest in COVID-19-related themes. It could also be the fact that some countries/regions have recently been planning to lift COVID-19 restrictions including mask wearing, such as Denmark and England (32), reflecting decreasing support to mask wearing at institutional level.

Though cloth masks provided less protection against COVID-19 than higher level masks such as N95, FFP2, or surgical masks (33), protective power would not be the sole consideration during choosing a mask. Other considerations included fashionability (e.g., of cloth masks) (34), level of comfort (35), and reservation of limited supply of N95 and surgical masks to healthcare workers (36). Moreover, through the course of the pandemic the different countries had different requirements (sometimes the same country switching the requirement from one to another type) for protective masks wearing. Also of importance for the interpretation of our data, the choice of mask is not the only influencing factor on the rate of COVID-19 transmission, as one needs to account for many other factors including virulence of the dominating strains and various social distancing measures. Regardless of mask type, the frequent use of masks has created environmental issues. Masks and hand gloves have become the commonest items among the personal protective equipment to be disposed of and pollute the environment (37). Although environmental pollution seems to be a less important problem during the pandemic, it could not be neglected and might influence the decision of some individuals or governments.

Finally, the reader should be aware that the current work has several limitations. First, Google Trends RSV reflected online searches through Google but not through other search engines such as Bing and Yahoo. Second, online searching for masks might not accurately reflect the actual behavior of or compliance to mask wearing. Third, supply might impact demand. At one point, China was contributing 50% of global mask production (38). To circumvent the supply shortage and save the medical masks for the healthcare workers in the initial phase of the COVID-19 pandemic, some countries might instruct the public to use cloth mask, such as the United States (39, 40). However, we did not have access to the market supply data on various mask types in the analyzed countries. From a sociocultural perspective, the decision of wearing or not wearing a mask might relate to individual perceptions of infection risk, personal interpretations of social responsibility, cultural traditions/religious practice, and the need to express self-identity (41). These factors might change over time and influence the online search for masks, but we could not evaluate them easily. In addition, the COVID-19 reproduction rate was a quantity that depended on not only population density but also spatial components such as movement, among other factors. This might partly explain the differences in the correlational results between RSV and reproduction rate vs. RSV and new cases per million (which mainly accounted for population density). Finally, the use of correlation tests could not distinguish between predictor and predicted variables (cause-effect relationship), see (42). Readers should be aware that the online interest for masks could be related to non-COVID-19 issues such as other airborne diseases. Complex statistical models might be applied to the data, but Pearson and Spearman correlation tests were the most frequently used statistical tests in studies using Google Trends data according to a recent systematic review (43). A cross-correlation test was subsequently performed to partially address this issue. These limitations should be taken in consideration for the results interpretation.

The following points could be concluded: (1) online searching for masks peaked during the first wave of COVID-19 pandemic and before the government implemented mandatory mask wearing for most of the surveyed countries; (2) it mostly positively correlated with government response stringency index but not COVID-19 reproduction rate or new cases per million; and (3) the population in different countries searched for different types of masks. The online searching behavior for masks varied markedly across countries with highest total number of COVID-19 cases.

## Data availability statement

The original contributions presented in the study are included in the article/Supplementary material, further inquiries can be directed to the corresponding authors.

## Author contributions

AY and AA conceived the study and wrote the first draft. AY collected the data. EP, JH, MK-P, OK, and HW critically revised the draft. All authors have approved the submitted version and have agreed to be accountable for the work.
