# Supplementary material for: Public interest in different types of masks and its relationship with pandemic and policy measures during the COVID-19 pandemic: a study using Google Trends data
Source: Front Public Health. 2023 Jun 8;11:1010674. doi: 10.3389/fpubh.2023.1010674 (PMC10286862; doi:10.3389/fpubh.2023.1010674)

Supplementary Figure 1. The search process through Google Trends with N95 mask as an example. First, N95 was typed into the search box. From the droplist, N95 could be searched as a custom “search term”, or as a predefined “topic” called “Particulate Respirator Type N95”. The latter was chosen. According to Google, a “topic” includes a group of terms that share the same concept. In the following webpage, the default “All categories” and “Web search” were chosen, whereas the date range was customized. Data was obtained for the “Worldwide”, and then the search was repeated for each country.


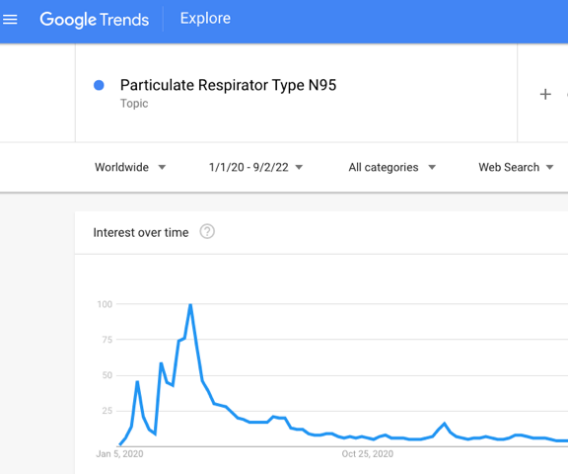


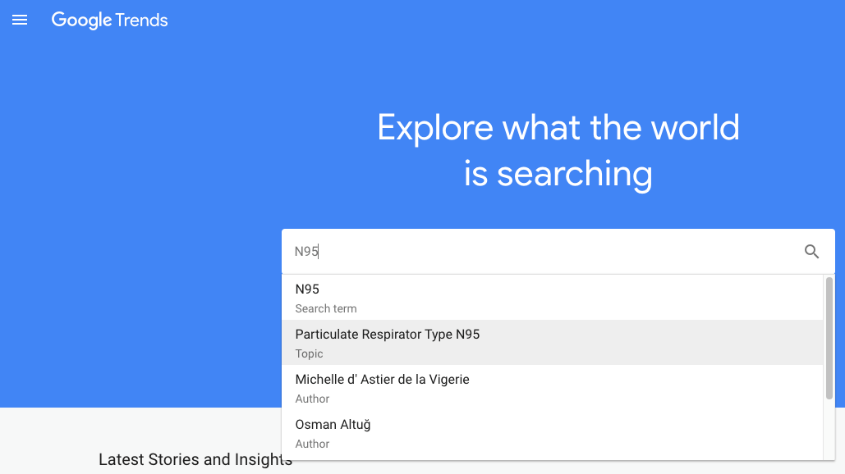

Supplement: Supplementary file 2 [file Data_Sheet_1.docx]
